# Development of an inhalable dry powder formulation for inhibition of SARS-CoV-2

**DOI:** 10.1016/j.ijpx.2025.100346

**Published:** 2025-06-14

**Authors:** Justin Stella, Anja Germann, Oliver Janka, Sylvia Wagner, Marc Schneider

**Affiliations:** a Department of Pharmacy, Biopharmaceutics and Pharmaceutical Technology, Saarland University, Campus C4 1, 66123 Saarbrücken, Germany; b Fraunhofer Institute for Biomedical Engineering IBMT, Department Bioprocessing & Bioanalytics, Joseph-von-Fraunhofer-Weg 1, 66280 Sulzbach, Germany; c Inorganic Solid State Chemistry, Saarland University, Campus C4 1, 66123 Saarbrücken, Germany

**Keywords:** Pulmonary application, Microparticle, Angiotensin converting enzyme 2, Serine protease inhibitor, Camostat, Co-amorphous formulation, Spray drying

## Abstract

Coronaviruses, including SARS-CoV-2, can cause significant lung damage and may result in multiple organ failure. The severity of COVID-19 is determined by the virus's entry into lung tissue and subsequent replication. This entry is facilitated by the angiotensin-converting enzyme 2 (ACE2) in combination with the serine protease TMPRSS2, which is a critical step. To reduce viral replication, it is necessary to prevent the uptake of the virus directly at the main route of transmission, which is the deposition of the virus as an aerosol in the respiratory tract. To reduce viral uptake into lung cells, an inhalable dry powder formulation was developed. The formulation contains camostat, a clinically proven serine protease inhibitor that inhibits the cellular uptake mechanisms on the lung surface. Camostat was spray-dried together with the mucolytic agent *N*-acetylcysteine to produce co-amorphous microparticles with sufficient solubility after deposition. Microparticles with properties suited for deposition in the deep part of the respiratory tract can be produced by using appropriate spray-drying parameters. The use of L-leucine enabled suitable aerodynamic properties and storage stability due to reduced interaction with environmental water. The geometric particle diameter, determined using laser light diffraction, decreased with L-leucine content which was found forming a partially crystalline L-leucine shell. The disintegration behavior of the microparticle formulation simulated under lung-like conditions indicated fast disintegration. A pseudo-viral *in vitro* assay demonstrated low acute toxicity in combination with a high activity. Cell viability and proliferation were not affected by camostat concentrations up to 11.1 μg/mL. The IC_50_ values of the two dry powder formulations tested on a HEK293T/ACE2-TMPRSS2 cell line were 0.008 μg/mL and 0.019 μg/mL, respectively, which is at least 100 times lower than the cytotoxic concentration. This dry powder formulation serves as a prototype microparticle matrix for incorporating nanoscale drug carriers in the future.

## Graphical abstract

**Figure:**
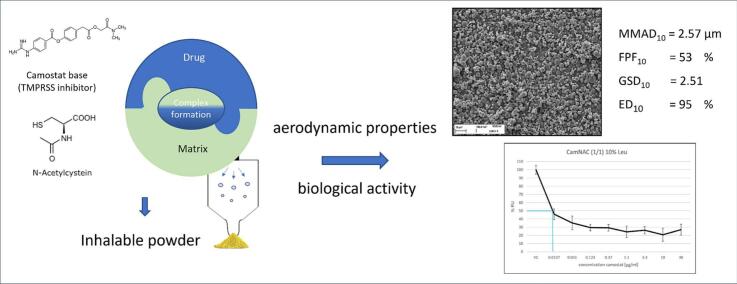

## 1 Introduction

The Coronavirus Disease 2019 (COVID-19) pandemic presented an unprecedented global health challenge caused by Severe Acute Respiratory Syndrome Coronavirus 2 (SARS-CoV-2), a novel betacoronavirus with a single-stranded RNA genome, belonging to the Coronaviridae family (Al-Rohaimi and Al Otaibi, 2020; Hoffmann et al., 2020). The virus targets the respiratory system, causing a range of clinical manifestations from asymptomatic or mild cases to severe pneumonia, acute respiratory distress syndrome (ARDS), and death (Al-Rohaimi and Al Otaibi, 2020; Beyerstedt et al., 2021).

SARS-CoV-2 enters host cells primarily through the angiotensin-converting enzyme 2 (ACE2) receptor, which is prominently expressed in the respiratory epithelium. The virus then undergoes replication, triggering a cascade of immune responses (Beyerstedt et al., 2021). The incubation period of COVID-19 can range from two to 14 days, and individuals can transmit the virus while asymptomatic, making it difficult to control its spread (Beyerstedt et al., 2021). COVID-19 presents a range of clinical severity, with common symptoms including fever, cough, and shortness of breath. In severe cases, the disease can progress to respiratory failure, cytokine storms, and multi-organ dysfunction (Al-Rohaimi and Al Otaibi, 2020).

In context of the coronavirus pandemic, standard inhaled medication such as corticosteroids and bronchodilators were utilized for the symptomatic treatment of COVID-19 for their potential to modulate the immune response and alleviate respiratory symptoms in COVID-19 patients (Bafadhel et al., 2022). The approach aims to deliver drugs directly to the site of infection in the deep lung (Schaller et al., 2020), potentially enhancing their efficacy. Therefore, several inhalation approaches were described and patented involving nebulized or sprayed formulations (Saha et al., 2022).

Thus, a dry powder formulation has been developed for this purpose, which contains the clinically proven serine protease inhibitor camostat and the mucolytic agent *N*-acetylcysteine (NAC). Camostat mesylate, marketed under the brand name ‘Foipan’, is an approved treatment for pancreatitis and postoperative reflux esophagitis (Ratan et al., 2023). The antiviral agent was frequently used off-label to alleviate COVID-19 symptoms during the global pandemic (McKee et al., 2020; Sakr et al., 2021) due to the fact that camostat has shown before efficacy *in vivo* and *in vitro* as antiviral agent (Hoffmann et al., 2021; Hoffmann et al., 2020; Zhou et al., 2015). However, a study by Gunst et al. for oral application of camostat did not indicate an improvement for patients treated if taken orally (Gunst et al., 2021). Camostat inhibits transmembrane protease serine 2 (TMPRSS2) activity, which is crucial in priming the viral spike protein (S protein) for the fusion of viral and cellular membranes (Hoffmann et al., 2020). By interrupting this process, camostat prevents the entry of SARS-CoV-2 into host cells with confidence (Hoffmann et al., 2020; Ragia and Manolopoulos, 2020). Camostat is addressing a specific pathway and other pathways might also be possible such as using aprotinin (Redondo-Calvo et al., 2022). Also alternatively, the use of neutralizing antibodies was considered (Chow et al., 2023). NAC exhibits antimicrobial properties by decreasing biofilm formation and extracellular polysaccharide matrix production. Its mucolytic effect is achieved by cleaving disulfide bonds in mucins, resulting in reduced mucus viscosity (Manoharan et al., 2020; Meziu et al., 2023). This is especially crucial for COVID-19 patients, who frequently experience increased mucus production and mucus viscosity as a symptom of the disease (Kato et al., 2022; Kumar et al., 2021). Furthermore, NAC exhibits anti-inflammatory properties by decreasing the levels of specific inflammatory mediators, such as tumor necrosis factor alpha (TNF-α) and certain interleukins, in COVID-19 patients (dos Santos Tenório et al., 2021; Zhou et al., 2020).

The objective of this study was to combine two active ingredients in a formulation using spray drying, due to their complementary properties. Spray drying follows the thermodynamic path of the production of amorphous drugs. Amorphous substances lack orientation or long-range order and are therefore physically unstable and can recrystallize (Wang et al., 2021). *Co*-amorphization can enhance the physical stability of amorphous drugs by formulating two low-molecular-weight crystalline drugs together as a non-polymeric glass solution or *co*-amorphous system (Liu et al., 2021). This approach not only increases physical stability but also enhances the solubility of the formulation (Wang et al., 2021).

Inhaled dry powder formulations have a special position among respiratory therapeutics due to their many advantages. It is important to note that these formulations should be used for delivering active ingredients directly to the lungs (AboulFotouh et al., 2020; Brunaugh and Smyth, 2018). They enable the targeted and localized administration of drugs to the respiratory system, resulting in a high concentration of active ingredients at the site of infection. This reduces systemic exposure and potential side effects while maximizing therapeutic efficacy (AboulFotouh et al., 2020) and might overcome the limitations for treatment described for oral camostat delivery (Gunst et al., 2021).

Dry powder formulations dissolve and absorb quickly, resulting in a rapid onset of action. This property is particularly advantageous in the treatment of acute respiratory infections, where rapid intervention is critical to halt the progression of the disease (AboulFotouh et al., 2020). Additionally, dry powder formulations often have better storage stability compared to liquid aerosols, reducing the risk of drug degradation during storage (Tran et al., 2020). However, the advantages of dry powder formulations are offset by several requirements that must be met for effective inhalation application. Optimal drug deposition in the respiratory tract necessitates careful control of particle size distribution. Fine particles with an aerodynamic diameter of 1 to 5 μm are ideal for deposition in the deep lung region, ensuring effective drug delivery (Benke et al., 2020). In this study, we first examined the particle size distribution and aerodynamic properties of the prepared dry powder formulations to estimate their flight and deposition behavior. Additionally, X-ray powder diffraction was used to characterize the solid formulation and test for amorphousness. The storage stability was also tested to observe any morphological changes, as spray-dried or amorphous drug formulations are prone to recrystallization or agglomeration. The disintegration behavior of the particles was evaluated under lung-like conditions, considering the limited volume of the dissolving medium in the lung. Finally, the antiviral efficacy and cytotoxicity of the formulation were assessed.

## 2 Materials and methods

### 2.1 Materials

Camostat mesylate was purchased from Natural Micron Pharm Tech (Shandong, China). *N*-acetylcysteine and sodium hydroxide solution were obtained from Carl Roth GmbH (Karlsruhe, Germany). L-leucine, and rhodamine B were provided by Sigma Aldrich (Steinheim, Germany). Brij 35® was supplied by Merck (Darmstadt, Germany), Highly purified and desalinated water was produced using the Milli-Q® Direct water treatment system from Merck (Darmstadt, Germany). A colorimetric immunoassay based on the measurement of Bromodeoxyuridine (BrdU) incorporation during DNA synthesis was used to quantify cell proliferation. A WST-1 assay (water soluble tetrazolium) was used to determine cell viability. Both assays were supplied by Merck (Darmstadt, Germany).

### 2.2 Resalting of camostat mesylate

For the spray drying process, camostat was used after removing the methanesulfonic acid. To achieve this, 0.1 M sodium hydroxide solution was added to an aqueous solution of camostat mesylate. The precipitated camostat was then centrifuged at 2000*g* for 10 min, and the supernatant containing dissolved methanesulfonic acid was removed. MilliQ water was added to the camostat, which was then centrifuged. This washing step was repeated. The camostat pellet was subsequently frozen at −80 °C and lyophilized.

### 2.3 Spray drying of the dry powder formulation

The dry powder formulations were prepared using a BÜCHI B-290 spray dryer (Flawil, Switzerland). Camostat and NAC were mixed in an equimolar ratio and then combined with L-leucine, which was gradually increased in amount (refer to Table 1). The addition of L-leucine reduces the cohesion of the spray-dried microparticles, improving their storage stability and aerodynamic properties (Alhajj et al., 2021; Rabbani and Seville, 2005). The mixing solution was adjusted to achieve a total concentration of 0.5% by mass for all solids for spray drying. To quantify, 100 μL of a 5 mg/mL aqueous solution of rhodamine B per 100 mg of dry substance was added to the spray drying solution. The inlet temperature of +55 °C resulted in an outlet temperature of +38 °C. The volumetric flow rate of the drying air was 35 m^3^/h and the air pressure at the spray nozzle was 1050 L/h. The feed solution had a flow rate of approximately 3 mL/min. All formulations were spray-dried with compressed air. The resulting powders were then collected and stored in a desiccator under vacuum at room temperature. Each formulation was prepared three times separately.

**Table 1:** Microparticle formulation consisting of equimolar amounts of camostat and NAC with increasing L-leucine content in mass percent (wt%).

Formulation	Camostat [wt%]	NAC [wt%]	L-Leucin [wt%]
CamNAC (1/1) 0% Leu	70.94	29.06	0.00
CamNAC (1/1) 5% Leu	67.40	27.60	5.00
CamNAC (1/1) 10% Leu	63.85	26.15	10.00
CamNAC (1/1) 15% Leu	60.30	24.70	15.00
CamNAC (1/1) 20% Leu	56.75	23.75	20.00
CamNAC (1/1) 25% Leu	53.21	21.79	25.00

### 2.4 Morphology of dry powder formulations

Morphological analysis was conducted using a scanning electron microscope EVO HD 15 (Zeiss, Jena, Germany). Each powder sample was applied to a single carbon disk and excess powder was removed with a rubber blower. A 10 nm gold layer was then coated onto the sample using a Quorum Q150R ES sputter coater (Laughton, UK). Imaging was done with an acceleration voltage of 5.0 kV.

### 2.5 Determination of particle size distribution

The spray-dried powder was analyzed by laser light diffraction using the Horiba Partica LA-950. In this procedure, the sample was dispersed by introducing an air stream to create an aerosol. Approximately 1000 mg of the microparticle formulation was introduced into the sample chamber and subjected to aspiration with an airflow set at 150 L/min. Particle size distribution was determined using Fraunhofer diffraction principles.

### 2.6 Analysis of aerodynamic properties

A Next Generation Impactor (NGI) (Copley Scientific, Nottingham, UK) was used to study the aerodynamic properties of the dry powder formulations. Prior to the experiment, a solution of 6 parts Brij 35, 34 parts ethanol and 60 parts glycerol were applied to each impactor dish. 100 μL of the solution was used for the small dishes and 200 μL for the large dishes. The solution was spread with a sponge and allowed to evaporate for 10 min. Then 10 mL of Milli-Q water was added to the pre-separator. For each experiment, a hard gelatin capsule (size 3) was filled with approximately 20 mg formulation. The air flow for the application was set to 60 L/min and controlled by an M1A flow meter (Copley Scientific, Nottingham, UK). The capsules were placed in a HandiHaler (Boehringer Ingelheim, Ingelheim, Germany) and pierced. Aerosolization of the powder was achieved by applying a gas flow for 4 s using a vacuum pump and flow controller (ERWEKA, Heusenstamm, Germany). Quantification of the powders deposited in the different NGI vessels was performed by dissolving them with a defined amount of water and quantifying the fluorescence signal of rhodamine B using a Tecan Infinite 200 reader (Tecan, Männedorf, Switzerland). An individual calibration curve was generated for each formulation and the entire formulation was analyzed at an excitation wavelength of 565 nm and an emission wavelength of 625 nm. Each formulation was run and measured three times.

The cumulative mass fractions were determined for all stages and converted to probit values. The probit values were then plotted as a function of the logarithmic cut-off diameter of each stage. Specific curve points were used for the linear regression, namely those with a logarithmic cut-off diameter of 0.7, which is relevant for the calculation of the fine particle fraction, and a probit of 5, which is required for the calculation of the mass median aerodynamic diameter (MMAD). The MMAD is defined as the diameter corresponding to a cumulative mass weighted fraction of 50% and therefore has a probit of 5 (Torge et al., 2019).

The geometric standard deviation (GSD) was calculated using the following equation:

$$\mathrm{GSD}={\left(d_{84}/d_{16}\right)}^{1/2}$$

d_84_ and d_16_ are defined as the diameters corresponding to the 84% and 16% percentiles, respectively, of the cumulative mass-weighted aerodynamic particle size distribution (Torge et al., 2019).

The fine particle fraction (FPF) was calculated by converting the corresponding probit for a cut-off diameter of 5 μm (corresponding to a logarithmic cut-off diameter of 0.7) into the corresponding cumulative mass fraction. The FPF represents the fraction of powder released from the capsule with an aerodynamic diameter less than 5 μm. Therefore, the mass of particles smaller than 5 μm determined in the NGI cups was related to the total mass of powder released from the capsule, including the powder in the cups, the pre-separator, and the inlet (Torge et al., 2019).

The emitted dose (ED) was defined as the mass of powder loaded into the capsule that was released during aerosolization, determined spectrometrically and expressed as a percentage of the total dose per capsule.

### 2.7 Solid-state characterization using an X-ray powder diffraction

Powder X-ray diffraction (PXRD) patterns of the powdered DPI formulations were recorded at room temperature on a D8-A25-Advance diffractometer (Bruker, Karlsruhe, Germany) in Bragg-Brentano *θ-θ*-geometry (goniometer radius 280 mm) with Cu *K*α_1,2_-radiation (λ_1_ = 154.0596 pm, λ_2_ = 154.4426 pm). A 12 μm Ni foil working as *K*β filter and a variable divergence slit were mounted at the primary beam side. A LYNXEYE detector with 192 channels was used at the secondary beam side. Experiments were carried out in a 2*θ* range of 6 to 130° with a step size of 0.013° and a total scan time of 1 h. After four weeks, the samples were measured again to check for crystallization.

### 2.8 Disintegration behavior under lung-like conditions

A 0.1% agarose solution was prepared by stirring agarose in hot water for 30 min. The solution was then poured into Petri dishes and left to cool overnight. The spray-dried powders were applied to a cellulose membrane using cotton swabs and spread evenly. The membrane was then placed on the agarose gel pad mimicking the moist lung surface. To simulate the humidity in the lungs, the samples were incubated for 5 min at a relative humidity of 100% and a temperature of +37 °C. Subsequently, the membranes were removed from the gel pads and analyzed using the 3D measurement Olympus OLS 4100 laser scanning microscope (Olympus Corporation, Tokio, Japan).

### 2.9 Morphological stability

The stability tests were conducted using an EVO HD 15 scanning electron microscope from Zeiss (Jena, Germany). Each dry powder formulation was divided into two batches during production. One batch was stored under vacuum in a desiccator with silica gel at room temperature (+20 °C), while the second batch was stored in a sealed container at room temperature. The storage conditions were selected to enable a comparison between formulations stored under optimal conditions (0% RH) and those stored under ambient conditions (40% RH). The formulations were analyzed for morphological changes immediately after production and again after two weeks using scanning electron microscopy.

### 2.10 Cell cultivation

All cell lines were incubated at +37 °C and 5% CO_2_ in a humidified atmosphere.

BHK-21 (G34) cell line was kindly provided by Stefan Pöhlmann (German Primate Center GmbH – Leibniz Institute for Primate Research, Göttingen, Germany). Cells were cultivated in Dulbecco's modified Eagle's medium (DMEM, high glucose) (Gibco, Schwerte, Germany) supplemented with 10% fetal bovine serum (Merck, Darmstadt, Germany) and 100 U/mL Penicillin, 100 μg/mL Streptomycin (Gibco, Schwerte, Germany). In every fourth passage, the medium was additionally supplemented with 100 μg/mL Zeocin and 50 μg/mL Hygromycin B (Gibco, Schwerte, Germany).

HEK293T/17 cells (ATCC® CRL-11268™) were obtained through LGC Promochem (Molsheim, France) and were cultivated in Dulbecco's modified Eagle's medium (DMEM, high glucose) (Gibco, Schwerte, Germany) supplemented with 10% fetal bovine serum (Merck, Darmstadt, Germany), 25 mM HEPES (Gibco, Schwerte, Germany) and 50 μg/mL Gentamicin (Gibco, Schwerte, Germany).

HEK293T/ACE2-TMPRSS2 cells, stably expressing angiotensin-converting enzyme 2 (ACE2) gene and transmembrane serine protease 2 (TMPRSS2) gene (HEK293T/ACE2-TMPRSS2) (GeneCopoeia Inc., Rockville, MD, USA) were cultivated in Dulbecco's modified Eagle's medium (DMEM, high glucose) (Gibco, Schwerte, Germany) supplemented with 10% fetal bovine serum (Merck, Darmstadt, Germany), 100 U/mL Penicillin, 100 μg/mL Streptomycin (Gibco, Schwerte, Germany), and 1 μg/mL Puromycin (Gibco, Schwerte, Germany).

### 2.11 Quantification of Cell viability

Using WST-1 assays (Sigma-Aldrich Chemie GmbH, Darmstadt, Germany) the cell viability was determined following treatment with serial 3-fold dilutions of camostat mesylate, CamNAC (1/1) 10% Leu and CamNAC (1/1) 20% Leu according to the manufacturer's protocol. In brief, HEK293T/ACE2-TMPRSS2 cells (3x10E4) were grown to subconfluency in 96-well microtiter plates and incubated for 24 ± 2 h at +37 °C with 5% CO_2_. Next, serial 3-fold dilutions of the samples were added to the cells, resulting in final concentrations between 0.14 μg/mL to 100 mg/mL. Wells containing only cell culture medium served as assay background control and 0.2% 2-hydroxyethyl methacrylate (Sigma-Aldrich Chemie GmbH, Darmstadt, Germany) as positive control (PC). After exposure of 48 ± 2 h, cell culture supernatants were aspirated. WST-1 solution (WST-1 reagent 1:11 diluted with cell culture medium) was added. Formazan absorption was measured at 450/650 nm after 100 min incubation using the Infinite F200 microplate reader (Tecan, Männedorf, Switzerland) which correlate with the activity of mitochondrial dehydrogenases in the cells.

Cell proliferation was measured using the Cell Proliferation ELISA assay (BrdU (colorimetric), (Roche Diagnostics, Mannheim, Germany) determined following treatment with serial 3-fold dilutions of camostat mesylate, CamNAC (1/1) 10% Leu and CamNAC (1/1) 20% Leu according to the manufacturer's protocol. In brief, cells were prepared and treated as described above. After 48 ± 2 h of the samples' exposure, cells were fixed. BrdU antibody solution (1:11 diluted with cell culture medium) was added and incubated for 90 min After addition of the substrate, cells were incubated for 4 min and reaction was stopped by 1 M H_2_SO_4_ (Sigma-Aldrich Chemie GmbH, Darmstadt, Germany). Absorption was measured at 450/650 nm after 100 min incubation using the Infinite F200 microplate reader (Tecan, Männedorf, Switzerland). The measured absorption values directly correlate to the amount of DNA synthesis and hereby to the number of proliferating cells.

The mean absorbance of untreated cells was defined as 100%, and the absorbance of the treated cells was related to this value. To avoid false positives effects in the virus inhibition assay, only sample concentrations with a cytotoxicity of less than 95% were selected for drug efficiency studies.

### 2.12 Antiviral inhibition

#### 2.12.1 Preparation of VSV*deltaG-FLuc particles

VSV*deltaG-FLuc basic stock was kindly provided by Gert Zimmer (Institute of Virology and Immunology, Mittelhäusern, Switzerland) and Stefan Pöhlmann (German Primate Center GmbH – Leibniz Institute for Primate Research, Göttingen, Germany). VSV*deltaG-FLuc are based on a replication-restricted vesicular stomatitis virus (VSV) that codes for an enhanced green fluorescent protein (eGFP) and firefly luciferase (FLuc) instead of the parental VSV glycoprotein (VSV-G) (Berger Rentsch and Zimmer, 2011). The basic stock was multiplied according to the protocol published by Becker et al. (Becker et al., 2021). In brief, BHK-21(G43) cells were grown to subconfluency in T75-flasks and incubated for 24 ± 2 h at +37 °C with 5% CO_2_. Cell culture medium was replaced by cell culture medium containing 0.01 μM Mifepristone (Sigma-Aldrich Chemie GmbH, Darmstadt, Germany) for 6 h. After removing the medium, VSV*deltaG-FLuc (1:1000 diluted in Dulbecco's modified Eagle's medium (DMEM), high glucose) was added to the cells and incubated for 1 h. Inoculum was removed, and cells were cultivated in culture medium containing 0.01 μM Mifepristone and 5% fetal calf serum. Supernatant containing VSV*deltaG-FLuc particles were removed, centrifuged and cryopreserved at −80 °C.

#### 2.12.2 Titration of VSV*delta G-FLuc

VSV*deltaG-FLuc particles were titrated using HEK293T/17 cells (Becker et al., 2021). In brief, HEK293T/17 cells (3 × 10^4^) were grown to subconfluency in 96-well microtiter plates for 24 ± 2 h at +37 °C with 5% CO_2_. Serial 10-fold dilutions of VSV*deltaG-FLuc were prepared. After removing the cell culture medium, cells were inoculated with serial virus particle dilutions (100 μL) and incubated for 1 h at +37 °C with 5% CO_2_. Cells were washed using Dulbecco's modified Eagle's medium (DMEM, high glucose). Cells were incubated for 24 ± 2 h at +37 °C with 5% CO_2_ to allow expression of eGFP. Wells containing 20–200 eGFP-positive cells were counted using fluorescence microscopy. Virus particle titer [ffu/mL] were calculated according to the following formula:

average number of eGFP-positive cells × reciprocal serial dilution factor (dilution in which eGFP-positive cells were counted) × inoculum dilution factor (10, since 100 μL were used for inoculation).

#### 2.12.3 SARS-CoV-2/VSV*deltaG-FLuc pseudovirus preparation

VSV*deltaG-FLuc particles were titrated using HEK293T/17 cells with some minor modifications (Becker et al., 2021). In brief, 7.5 × 10^5^ HEK293T/17 cells were grown to subconfluency in 6-well plates and incubated for 24 ± 2 h at +37 °C with 5% CO_2_. Transfection: transfection mix was prepared using Fugene HD (Promega GmbH, Walldorf, Germany) and 8 μg Spike protein expression plasmid (pCG1-SARS-CoV-2-S, kindly provided by Stefan Pöhlmann (German Primate Center GmbH – Leibniz Institute for Primate Research, Göttingen, Germany) and incubated to allow complex formation for 30 min at room temperature. Cell culture medium was removed, and transfection complex was added to the cells followed by 6 h incubation at +37 °C with 5% CO_2_. After removing the transfection complex, fresh culture medium was added, and cells were incubated for 20 ± 2 h at +37 °C with 5% CO_2_. Transduction and neutralization: cell culture medium was removed, and cells were inoculated with 1 mL VSV*deltaG-FLuc [multiplicity of infection =3]. After incubation for 1 h at +37 °C and 5% CO_2_, inoculum was removed, and cells were washed with DMEM (high glucose). Cell culture medium was added containing 16 ng/mL anti-VSV-G antibody [8G5F11] (Biozol, Eching, Germany) followed by incubation for 20 ± 2 h at +37 °C with 5% CO_2_. Supernatant, containing SARS-CoV-2/VSV*deltaG-FLuc pseudotyped virus particles, were harvested, centrifuged, and cryopreserved at −80 °C.

#### 2.12.4 Titration SARS-CoV-2/ VSV*deltaG-FLuc pseudotyped virus

HEK293T/ACE2-TMPRSS2 cells (2 × 10^5^) were grown to subconfluency in 96-well microtiter plates and incubated for 24 ± 2 h at +37 °C with 5% CO_2_. Serial 5-fold dilutions of SARS-CoV-2/ VSV*deltaG-FLuc, containing 2.5 μg/mL Polybrene (Nucleus Biotech GmbH Business Development Center Heidelberg, Germany), were prepared and added to the cells. Infected cells were incubated for 48 ± 2 h at +37 °C with 5% CO_2_ to allow expression of eGFP. Wells showing 20–200 eGFP-positive cells were counted using fluorescence microscopy. Virus particle titer [ffu/mL] were calculated according to the following formula:

average number of eGFP-positive cells × reciprocal serial dilution factor (dilution in which eGFP-positive cells were counted) × inoculum dilution factor (10, since 100 μL were used for inoculation).

#### 2.12.5 Drug efficiency study

HEK293T/ACE2-TMPRSS2 cells (3 × 10^5^, 100 μL) were grown to subconfluency in 96-well microtiter plates and were incubated for 24 ± 2 h at +37 °C with 5% CO_2_. Serial 3-fold dilutions of camostat mesylate (0.02–49.5 μg/mL), CamNAC (1/1) 10% Leu (0.0137–30 μg/mL) and CamNAC (1/1) 20% Leu (0.0046–10 μg/mL) were prepared, added to the cells, and incubated for 2 h at +37 °C with 5% CO_2_. SARS-CoV-2/ VSV*deltaG-FLuc particles [multiplicity of infection = 0.005], 2.5 μg/mL Polybrene and, if indicated camostat mesylate (0.02–49.5 μg/mL), CamNAC (1/1) 10% Leu (0.0137–30 μg/mL) and CamNAC (1/1) 20% Leu (0.0046–10 μg/mL), was added, and the cells were incubated for 48 ± 2 h to allow expression of eGFP. eGFP positive cells were used to calculate fluorescence forming units per mL (ffU/mL). Virus control (VC) ffU/mL value was set as 100% ffU and relative inhibitory concentrations of camostat mesylate, CamNAC (1/1) 10% Leu and CamNAC (1/1) 20% Leu were calculated as a function of virus control. Half maximal inhibitory concentrations (IC_50_) were calculated on the basis of the single-logarithmic slope between the measuring points above and below 50% ffU of the substance concentrations.

## 3 Results and discussion

### 3.1 Spray drying and morphological consideration

Spray drying is an attractive approach for obtaining micronized drug particles suitable for inhalation. Camostat, the active ingredient for inhibition of viral uptake into the lung cells, is poorly soluble in water and is therefore therapeutically used as water-soluble salt of methanesulfonic acid (camostat mesylate). To overcome the solubility issues the microparticle matrix can be formed by using forming a salt bridge with a second compound (Günday Türeli et al., 2016; Lababidi et al., 2020; Lababidi et al., 2019). Specifically, *N*-acetylcysteine (NAC) was used beneficial for spray drying but which is also described for treatment in COVID-19 (Alam et al., 2023; Avdeev et al., 2022; Ibrahim et al., 2020; Shi and Puyo, 2020). In addition, its mucolytic effect (Aldini et al., 2018; Manoharan et al., 2020; Meziu et al., 2023) and antimicrobial properties reducing biofilm formation and extracellular polysaccharide matrix production (Blasi et al., 2016) will help with bacterial infections associated with COVID-19 (Blasi et al., 2016; Cazzola et al., 2021; Wong et al., 2021). It reduces serum levels of certain inflammatory mediators, such as TNF-α and some interleukins, in COVID-19 patients (dos Santos Tenório et al., 2021; Zhou et al., 2020).

**Fig. 1:**
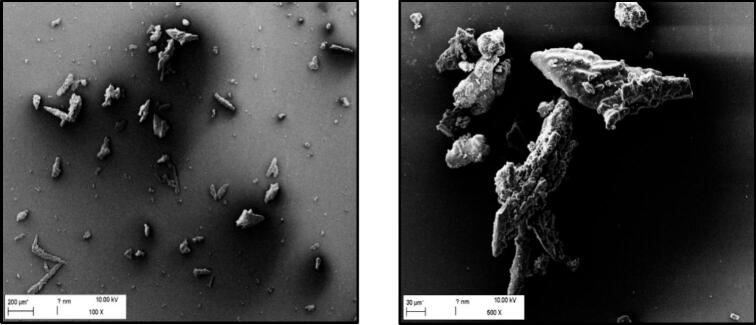
SEM image of spray-dried microparticles of camostat mesylate and *N*-acetylcysteine (equim.).

Initially, equimolar quantities of camostat mesylate and NAC were dissolved in water and spray dried. However, the product was sticky and difficult to harvest, resulting in a yield of only approximately 24% of the solid mass used and agglomerated particles (Fig. 1).

The images show elongated, sharp-edged, and coarse particles of the two active ingredients. The particle size varies between a few micrometers and approx. 250 μm. No further characterization of the product was performed as it can be assumed that it lacks sufficient aerodynamic properties. The product's appearance may be attributed to the hygroscopic nature of NAC (Gangopadhyay et al., 2020) and the inadequate interaction between the two active ingredients to form a salt (Walsh et al., 2018). Usually, dissolved substances dry simultaneously in the dispersed solvent droplet, which normally results in spherical microparticles. However, the hygroscopicity of the NAC causes the particles to stick together during or after the process due to the water content. This hypothesis is supported by the fact that the main part of the product consisted of a sticky film as mentioned above.

**Fig. 2:**
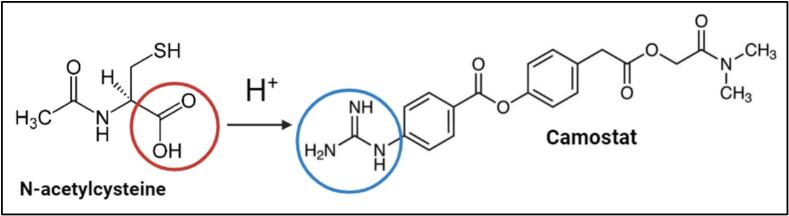
Protonation of the guanidine moiety of camostat by the carboxylic acid moiety of NACs.

**Fig. 3:**
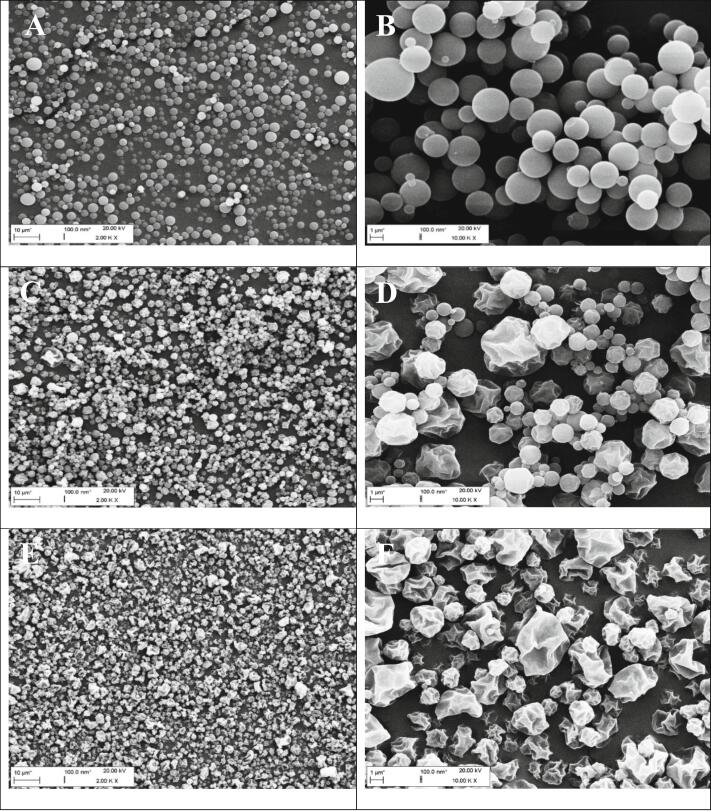
SEM micrographs show spray-dried microparticles with varying L-leucine content: microparticles without leucine (A + B), microparticles with 10% L-leucine and 90% camostat/NAC (equimolar) (C + D), and microparticles with 25% L-leucine and 75% camostat/NAC (equimolar) (E + F). Scale bars left column are 10 μm, for the right column 1 μm.

Transferring camostat into the base (Walsh et al., 2018) allows subsequent interaction between NAC and camostat. As a result, NAC can now protonate the guanidine function of camostat at the site of the methanesulfonic acid. The now negatively charged deprotonated carboxylic acid moiety of NAC can interact with the positively charged protonated guanidine moiety of the camostat via non-specific electrostatic interactions (Fig. 2) (Jensen et al., 2016). This led to a very fine white powder which was easy to collect and was showing spherical particles (Fig. 3A).

Estimation of salt formation can be made by comparing the pKa values of an acidic and a basic compound. If the difference is greater than 2, it is generally assumed that salt formation has occurred (Guillory, 2003; Serajuddin, 2007). The pKa of the carboxylic acid moiety of NAC is 3.84, while the pKa of the guanidine moiety of camostat is 8.50 (Guillory, 2003; Serajuddin, 2007). Since the difference is 4.66, this strongly suggests that salt formation has occurred between the two compounds.

L-leucine is well described to improve the properties of dry powder formulations for inhalation by shielding dry powders from moisture, preventing agglomeration and premature decomposition (Li et al., 2016). The butyl group of L-leucine enhances the hydrophobic properties of the formulation and prevents water from adsorbing on the particle surface and penetrating into deeper layers of the particles (Alhajj et al., 2021; Li et al., 2016; Mangal et al., 2015). During spray drying, L-leucine accumulates on the droplet surface, forming a hydrophobic crystalline outer shell that alters surface composition and morphology and reduces cohesive forces between different entities (Alhajj et al., 2021; Li et al., 2016; Mangal et al., 2015). However, the concentration of L-leucine needed to achieve this effect varies and must be analyzed on a case-by-case basis, depending on the properties of the drug (Alhajj et al., 2021).

All spray-dried dry powder formulations exhibited similar handling characteristics for collection and storage. The spray-dried product was easy to collect and did not show any visible agglomerates, indicating low moisture content and a low tendency to aggregate. Fig. 3 shows that all formulations consisted mainly of spherical particles, with an increasingly corrugated structure as the proportion of L-leucine increased. The particles without L-leucine have a smooth and perfectly spherical structure, as shown in Fig. 3A/B. No sign of strong agglomeration was observed. When comparing Fig. 3A and C, it is evident that the microparticle formulation with a 10% leucine content (Fig. 3C) is more homogeneously distributed than the leucine-free formulation (Fig. 3A). This can be attributed to the reduced interaction forces between objects due to the presence of L-leucine. Fig. 3D illustrates that the particles exhibit varying degrees of corrugation depending on their size, with larger particles being considerably more corrugated than smaller ones. The concentration of the two active ingredients in the center of the shrinking particle and the collection of hydrophobic L-leucine in the outer layer or at the interface with the gas phase during particle drying explains this phenomenon. Larger dispersed liquid droplets shrink faster and more strongly due to their surface or interaction area with the hot gas medium growing disproportionately to the diameter (Alhajj et al., 2021). If the spherical droplet diameter doubles, its surface area quadruples (O = π × d^2^). As the particle shrinks, the L-leucine shell collapses, resulting in the raisin-shaped appearance (Alhajj et al., 2021). Comparing Fig. 3C and D, the distribution density on the surface of the sample holder increases. Furthermore, Fig. 3B and D demonstrate that particle arrangement becomes less vertical and more horizontal indicating less adhesion and better flowability with increasing leucine content. However, the distribution density of the particles containing 25% leucine is visually only slightly higher than that of the particles containing 10% leucine. At the highest leucine concentration, all particles seem equally corrugated, regardless of their size.

### 3.2 Determination of particle size distribution

**Fig. 4:**
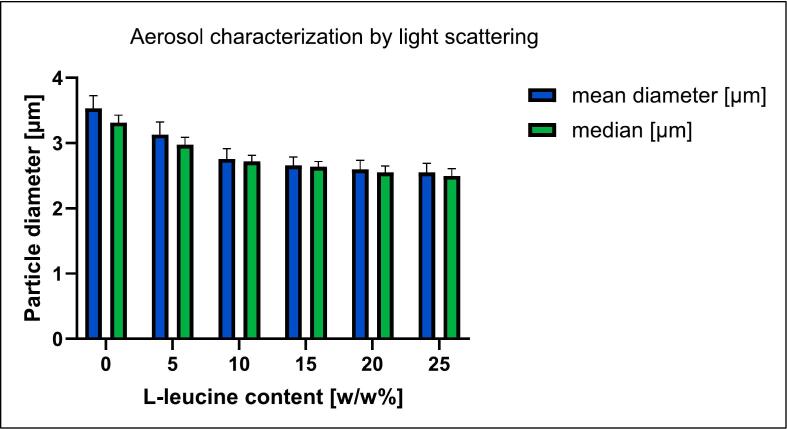
Mean and median particle diameters of spray-dried products were plotted against L-leucine content.

For a full characterization of the formulation, microparticles were analyzed for their sizes using light scattering. Target sizes should be around 1–5 μm as no very small densities were expected. The size distribution of the particles was determined using the Horiba Partica LA-950 laser diffractometer. Fig. 4 shows the mean and median particle diameters as a function of the percentage of used L-leucine. As particles form rapidly during spray drying, the resulting particle size distribution typically exhibits a log-normal shape (Elversson et al., 2003). Using the median instead of the mean is more robust against statistical outliers and provides a more accurate representation of the results, particularly when dealing with non-normally distributed data (Elversson et al., 2003).

The formulation without leucine has the largest particle diameters, with a mean diameter of 3.51 μm and a median value of 3.31 μm. The addition of small amounts of L-leucine, e.g., 10 wt%, initially decreases the mean and median particle diameter sharply to 2.75 μm and 2.71 μm respectively. Further increases in the amount of L-leucine have only a weak effect on the measured particle diameter. The median and mean diameter of the sample with 25% L-leucine are still 2.55 and 2.50 μm respectively. There are two reasons for the reduction in particle diameter with increasing leucine content. Firstly, due to its amphiphilic structure, L-leucine has surface-active properties that reduce the surface tension of the droplets. This leads to the formation of smaller droplets when the active ingredient solution is atomized, resulting in smaller microparticles (Ziaee et al., 2019). Secondly, L-leucine reduces the interparticle forces by forming a hydrophobic shell (Mangal et al., 2015). This results in a reduction of agglomerates, leading to smaller particle diameters (mean and median). Due to the higher sensitivity to statistical outliers the mean of formulations with lower L-leucine content deviate more from the median as for formulations with larger L-leucin fraction. The convergence of the mean and median shown in Fig. 4 with increasing L-leucine content therefore means fewer statistical outliers *i.e.* less agglomerates. The standard deviations of both variables also decrease in this process. This indicates an increase in the reproducibility of the results.

The distribution of particle sizes is reflected by the 10th and 90th percentiles and the resulting span value (Table 2). If we first compare the values of the 90th percentile, as expected, it is highest for the formulation without L-leucine and lowest for the formulation with 25% L-leucine. This is consistent with previous observations indicating a reduction in mean or median particle diameter at high L-leucine levels. Except for the leucine-free formulation, all values of the 90% percentile are below 5 μm, *i.e.* 90% of the particle collective is potentially suited for deep lung deposition. The values of the 10% percentile also decrease with increasing L-leucine content but are all above 1 μm. Inhalation of microparticles smaller than 0.5 μm increases the risk that they will not deposit and follow the exhalation stream (Artmann and Staudinger, 2021; Bake et al., 2019). However, the majority of particles in the dry formulations produced are larger, so this risk is negligible. Finally, a comparison of the range, which is calculated from the two percentiles and the median indicates the width of the particle size distribution, shows that all values are between 1.10 and 1.40. This indicates a very narrow and a monomodal particle size distribution (Simon et al., 2016). A narrow size distribution facilitates a targeted deposition behavior of the particles in the lung, which is especially relevant for very potent or side-effect-prone drugs (Islam et al., 2020). With a d50 between 2.50 and 3.32 μm, most spray-dried particles should be able to reach deeper areas of the lung, such as the bronchial and alveolar regions (Williams et al., 2011).

**Table 2:** Results from static light scattering for the particles' size distribution of the six spray-dried dry powder formulations consisting of a camostat and NAC in equimolar ratio, plus an increasing proportion of L-leucine.

Sample composition	Mean size (μm)	d10 (μm)	d50 (μm)	d90 (μm)	Span
CamNAC (1/1) 0% Leu	3.54	2.11	3.32	5.84	1.23
CamNAC (1/1) 5% Leu	3.13	1.74	2.98	4.95	1.10
CamNAC (1/1) 10% Leu	2.76	1.66	2.72	4.86	1.18
CamNAC (1/1) 15% Leu	2.65	1.35	2.64	4.65	1.25
CamNAC (1/1) 20% Leu	2.60	1.27	2.55	4.77	1.37
CamNAC (1/1) 25% Leu	2.56	1.11	2.50	4.62	1.40

In addition to the geometric particle diameter, the density and shape of the particles also have a strong influence on the aerodynamic properties of the particles. L-leucine not only affects the shape of the particles (Fig. 3 A to F), but also reduces the density of the spray-dried microparticles (Alhajj et al., 2021). To take these two factors into account, the dry powder formulations must also be tested for their aerodynamic behavior as the key parameter for pulmonary deposition.

### 3.3 Determination of Aerodynamic Properties

The efficacy of a dosage form for inhalation is heavily influenced by the aerodynamic properties of the powder particles. These properties affect the deposition behavior of the particles and subsequently the local drug concentration.

To analyze these properties NGI experiments were conducted under standard conditions, which included a specific gas flow rate and a fixed inhalation volume.

Studies have shown that particles with a mass median aerodynamic diameter (MMAD) of 5 to 10 μm are deposited primarily in large and medium conducting airways and in the oropharynx (Artmann and Staudinger, 2021). An MMAD of 1 to 5 μm is associated with deposition in the small airways and in the alveolar region. Specifically, particles with an MMAD smaller than 3 μm are deposited in the alveoli (Artmann and Staudinger, 2021). It is important to note that the MMAD values determined in this study may not only represent the MMAD values of the primary particles, but also of particle aggregates due to powder agglomeration and non-sufficient dispersion. The distribution width of the aerodynamic diameter is described by the geometric standard deviation (GSD). In pharmaceutical formulations, polydisperse aerosols are more common, as indicated by a GSD above 1.25 (Dechraksa et al., 2020).

The aerodynamic properties of the camostat/NAC powders as determined by the NGI are displayed in Table 2. The dry powder formulation without L-leucine has the largest MMAD with 3.46 μm. The addition of small amounts of L-leucine (5–10 wt%) initially decreases the MMAD, but this effect is only slight with further addition of leucine similar to the geometric sizes measured by light scattering. With an addition of approximately 15 wt% L-leucine, the MMAD no longer decreases. A comparison of the GSD values, i.e. the particle size distributions of the aerodynamic diameters, shows a slight but steady decrease with an increasing L-leucine content, from 2.71 to 2.23. This indicates that L-leucine contributes positively to targeted drug deposition by reducing the particle size distribution (Alhajj et al., 2021; Mangal et al., 2015). The fine particle fraction is lowest in the dry powder formulation without L-leucine at 27.9% and increases considerably to 45.9% with an application of 5 wt%. At 10% L-leucine, saturation of the fine particle fraction is already achieved. This may be due to the fact that the maximum reduction in interparticle cohesion occurs at around 10–‐15% L-leucine content as often observed also by others (Alhajj et al., 2021; Li et al., 2016).

The objective was to deliver the dry powder formulation to the alveolar region of the lung, which requires particles with an MMAD of less than 3 μm (Labiris and Dolovich, 2003). All dry powder formulations met this requirement except for the leucine-free formulation, which may have agglomerated due to the hygroscopic properties of the NAC. The GSD values, ranging from 2.23 to 2.71, indicate a polydisperse particle population. Considering these values most of the particles tested would deposit in the bronchial and alveolar region and only a small proportion is assumed to be deposited in the extrathoracic region (Artmann and Staudinger, 2021).

The emitted dose (ED) in Table 3 is the mass of capsule contents released during the aerosolization process expressed as percentage of the total dose per capsule. The emitted dose is already high with the leucine-free formulation and increased with increasing L-leucine content in the dry powder formulation. The improvement in the emitted dose can be explained by the above-mentioned reduction in interparticle cohesive forces, which leads to more efficient aerosolization of the particles (Li et al., 2016; Mangal et al., 2015).

**Table 3:** Aerodynamic properties: MMAD (Mass Median Aerodynamic Diameter), GSD (Geometric Standard Deviation), FPF (Fine Particle Fraction), the dry powder formulation of equimolar parts camostat and NAC, as well as an increasing L-leucine content (0– ‐ 25% (*w*/w). Each dry powder formulation was produced in three batches, with the results presented as mean values + standard deviation (triplicate determination).

	MMAD [μm]	GSD	FPF [%]	Emitted Dose [%]
CaNAC (1/1) 0% Leu	3.56 ± 0.22	2.71 ± 0.09	27.9 ± 2.4	88.76 ± 2.16
CaNAC (1/1) 5% Leu	2.89 ± 0.13	2.65 ± 0.10	45.9 ± 1.4	92.14 ± 1.61
CaNAC (1/1) 10% Leu	2.57 ± 0.10	2.51 ± 0.05	53.1 ± 3.7	95.26 ± 1.75
CaNAC (1/1) 15% Leu	2.46 ± 0.17	2.41 ± 0.07	56.2 ± 4.2	95.73 ± 1.42
CaNAC (1/1) 20% Leu	2.42 ± 0.19	2.36 ± 0.07	57.5 ± 2.3	96.55 ± 0.83
CaNAC (1/1) 25% Leu	2.39 ± 0.08	2.23 ± 0.06	58.0 ± 4.2	96.42 ± 0.72

### 3.4 Powder X-ray diffraction for determination of the solid structure

The diffractogram of an amorphous material exhibits broad and diffuse reflections at best instead of sharp, discrete diffraction maxima, as seen for crystalline materials (Lamy et al., 2019).

**Fig. 5:**
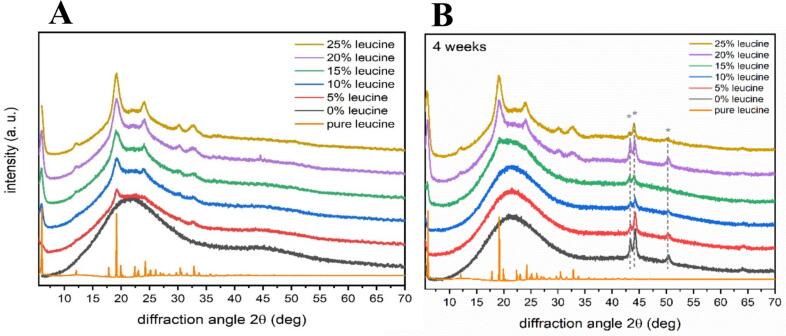
Diffractogram of pure L-leucine and the six spray-dried dry powder formulations with increasing L-leucine content. A) Measurements directly after preparation and B) Measurement after 4 weeks of storage in the desiccator.

The diffractograms of the six spray-dried dry powder formulations were determined for diffraction angles between 6 and 130°. Since the diffraction patterns show no reflections >70° 2*θ*, this region was omitted. Additionally, the diffraction pattern for pure L-leucine was determined yielding, as expected, sharp reflections typical for a crystalline substance (Fig. 5). In contrast, the dry powder formulations of camostat and NAC, free of leucine, exhibit a broad and diffuse diffraction pattern, typical for an amorphous solid. When compared to the diffraction patterns of the leucine-containing samples, it is noticeable that these show strongly broadened reflections, with maxima that coincide with the diffraction maxima of pure leucine. As the leucine content in the samples increases, the intensity of these maxima also increases. This indicates an increase in the total amount of crystalline leucine. However, no quantitative statements can be made about the amount of L-leucine. The results indicate that the components of the sample remain amorphous, regardless of the leucine content. Nano-crystalline L-leucine can be detected with an addition of only 5%. This suggests that the L-leucine shell forms somehow crystalline completely or partially even at low L-leucine concentrations. This may explain why positive effects on the aerodynamic properties occur even at very low L-leucine concentrations. After four weeks of storage in a vacuum desiccator with silica gel to avoid humidity, the amorphous character of the sample is still evident. The only changes are the with star (*)-marked peaks which are representing elemental iron. This is due to the very thin layer thickness of our sample resulting in the measurement of the underlying sample holder.

### 3.5 Determination of disintegration behavior under pulmonary conditions

The investigation of inhaled powder particle disintegration under lung-like conditions is crucial for developing effective and safe dry powder formulations. The disintegration of the formulation plays a key role, particularly when high humidity or low solvent volume is present in the lungs (Zillen et al., 2021). Inhaled powder particles must overcome specific challenges under lung-like conditions to ensure optimal drug release. The dissolution behavior of the microparticles directly affects the release of the active ingredient. Efficient dissolution allows for faster absorption of the drug by the lung tissue, resulting in a rapid effect. Optimal dissolution behavior requires a homogeneous distribution of the active ingredient. This is crucial to achieve a consistent effect on the target tissue and to avoid undesirable local over- or underdosing (Zillen et al., 2021). The dissolution profile of the particles is also important to avoid airway blockages and deposits which will also be crucial for onset of action and the acceptance by the patient (Labiris and Dolovich, 2003).

**Fig. 6:**
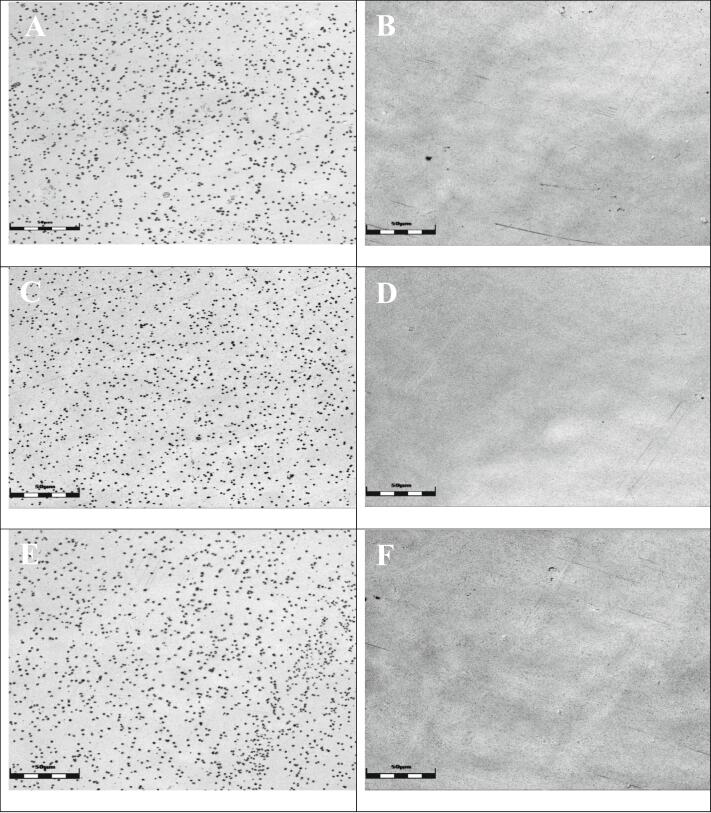
Visualization of the dissolution behavior under lung-like conditions: Before incubation: A) 0% L-leucine and 100% camostat/NAC (equim.), C) 10% L-leucine and 90% camostat/NAC (equim.), E) 20% L-leucine and 80% camostat/NAC (equim.); After incubation: B) 0% L-leucine and 100% camostat/NAC (equim.), D) 10% L-leucine and 90% camostat/NAC (equim.), F) 20% L-leucine and 80% camostat/NAC (equim.).Scale bars: 50 μm.

As previously stated, all dry powder formulations were applied directly onto the filter membrane. Prior to incubation, a white-light laser scanning microscope was used to examine the distribution of microparticles on the membrane surface (Fig. 6). Figs. 6A, C, and E demonstrate that the particles in all three samples are evenly distributed and well-separated from one another. After an incubation period of 5 min under lung-like conditions (100% RH, +37 °C, on a gel matrix), the filter membranes were removed from the gel pad. In Fig. 6 B, D, and F, the microparticles of the dry powder formulation are no longer visible as they have completely dissolved. Additionally, no drying residues of the particles can be observed, as the filter membrane allows absorption of the dissolved microparticle matrix during the disintegration process to avoid drying artefacts. So, the combination of the pure base camostat with NAC provides a fast-dissolving matrix. This is most likely also enhanced by L-leucine as reported in literature (Alhajj et al., 2021; Li et al., 2016; Mangal et al., 2015).

### 3.6 Determination of morphological stability

The stability of dry powder formulations for inhalation is a critical factor that affects the effectiveness and quality of this dosage form. Humidity significantly affects various properties of the powder particles. Efficient inhalation depends on the ability of the particles to disperse in the respiratory flow. High humidity can reduce the dispersibility of powder particles, causing them to stick together and making correct dosing more difficult. This can impair the aerodynamic properties and dispersion of the active ingredient (Li et al., 2016) and thus the homogeneous distribution of the active ingredient in the lungs. The formation of active ingredient agglomerates reduces the total surface area of the particles and, therefore, also reduces the interaction surface with the dissolution medium negatively effecting dissolution (Iwao et al., 2013).

**Fig. 7:**
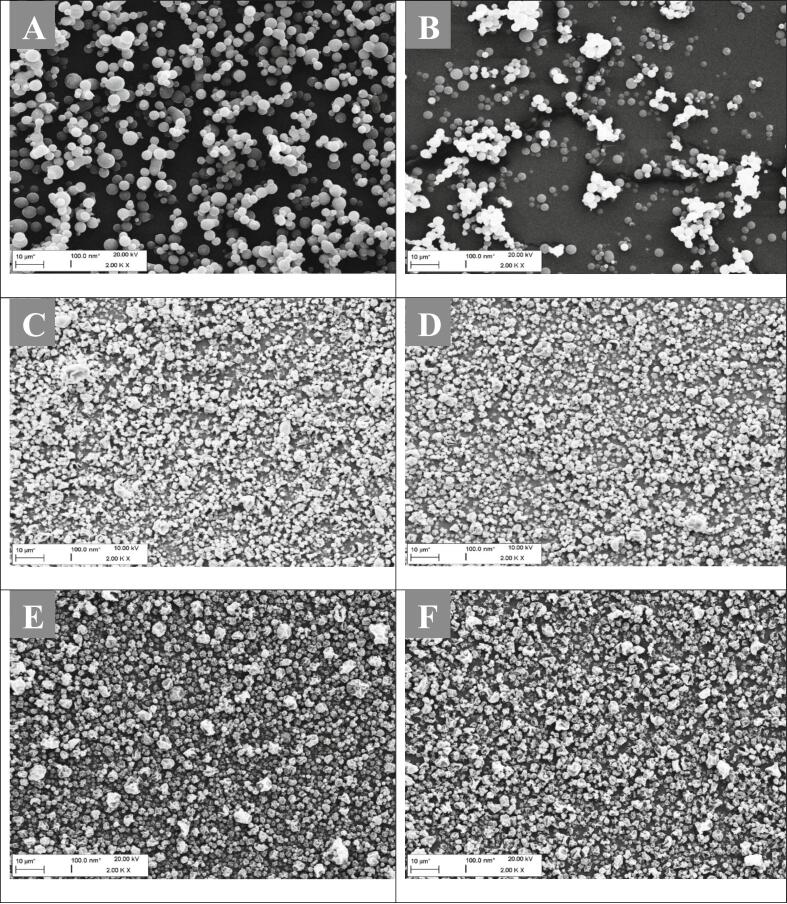
SEM micrographs of spray-dried samples after two weeks of storage: In desiccator: A) 0% L-leucine and 100% camostat/NAC (equim.), C) 10% L-leucine and 90% camostat/NAC (equim.), E) 20% L-leucine and 80% camostat/NAC (equim.); Under ambient conditions: B) 0% L-leucine and 100% camostat/NAC (equim.), D) 10% L-leucine and 90% camostat/NAC (equim.), F) 20% L-leucine and 80% camostat/NAC (equim.). Scale bar: 10 μm.

The dry powder formulations depicted in Fig. 7 were stored for a period of two weeks under two distinct storage conditions. The samples in Figs. 7A, C and E were stored in a desiccator with 0% relative humidity, while those in Figs. 7B, D and F were stored under ambient conditions with 40% relative humidity. The container lids were sealed airtight, meaning the contained powder could only interact with the air present in the container. This reflects more realistic conditions, as individual doses in a single-dose system are either individually blister-packed airtight (e.g., Diskus inhaler) (Iwao et al., 2013) or the multi-dose container of the dry powder inhaler (e.g., Turbohaler) is itself sealed airtight. In this case, we have a single dose stored and administered via a gelatine capsule. These capsules are typically stored either individually in blister packs or in a container with a desiccant additive (Shetty et al., 2020).

When comparing the leucine-free samples, it is evident that the sample stored in the desiccator agglomerates less than the sample stored under ambient conditions in a sealed container. This outcome was expected since the dry powder formulation does not interact with humidity in the desiccator. It is possible that the microparticles have already absorbed moisture on the surface during the time between collection and storage of the product. Both factors could be potential sources of low agglomeration (Fig. 7A). However, the sealed product is in contact with the air in the container. Fig. 7B shows agglomeration of particles in the 10 wt% leucine sample, which consists of characteristic raisin-shaped microparticles forming piles of particles. Despite different storage conditions, the two samples appear very similar and show no signs of agglomeration. The microparticles' L-leucine shell effectively reduces interparticle binding forces and the particles' interaction with air humidity. The dry powder formulation with the highest leucine content (20 wt%) also shows no signs of agglomeration and is distributed without indicating particle accumulation as for the 10 wt% leucine sample. A concentration of 10% by mass appears to be adequate for safeguarding the microparticle formulation against external factors, such as humidity, and for ensuring a uniform distribution of particles.

### 3.7 Cell viability

**Fig. 8:**
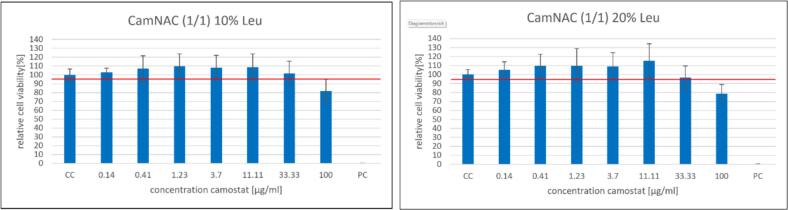
The graphs illustrate the results of a WST-1 assay to determine cell viability of the two dry powder formulations at different concentrations. The red line indicates 95% cell viability compared to the cell control. Left: CamNAC (1/1) 10% Leu composed of 63.85% camostat, 26.15% NAC and 10% L-leucine; right: CamNAC (1/1) 20% Leu composed of 56.75% camostat, 23.75% NAC and 20% L-leucine. (For interpretation of the references to colour in this figure legend, the reader is referred to the web version of this article.)

Besides antiviral efficacy, the two dry powder formulations containing 10 and 20% L-leucine were also tested for cell viability. Camostat concentrations in both dry powder formulations ranging from 0.14 to 100 μg/mL were used. Both graphs (Fig. 8) show that the cell viability of the formulations remains above 95% of the untreated cell control up to a camostat concentration of 33.3 μg/mL. Even at the highest tested camostat concentration of 100 μg/mL, the cell viability of both formulations is around 80%. The IC_50_ values of the two formulations are 0.008 μg/mL and 0.019 μg/mL, respectively. This indicates that the effective concentration is well below the cytotoxic concentration of 100 μg/mL, at which an effect on cell viability is observed. It is recommended to avoid a concentration of 100 μg/mL and ensure a reasonable dosage and distribution of the particles during inhalation. Due to the low IC_50_ values of camostat in its dry powder formulation, it is possible to reduce the amount of active ingredient or the total amount of dry powder per dose. The two graphs do not differentiate but show similar values across the entire concentration range. As the two formulations differ only in L-leucine content, the higher concentration of L-leucine also has no negative impact on cell viability in the WST-1 assay.

### 3.8 Cell proliferation

In addition to measuring cell viability, we also measured relative cell proliferation to determine the cytotoxicity of the dry powder formulations. We used an immunoassay to detect BrdU incorporation during DNA synthesis. Camostat concentrations in both dry powder formulations ranging from 0.14 to 100 μg/mL used for determining cell viability were also used for this purpose.

**Fig. 9:**
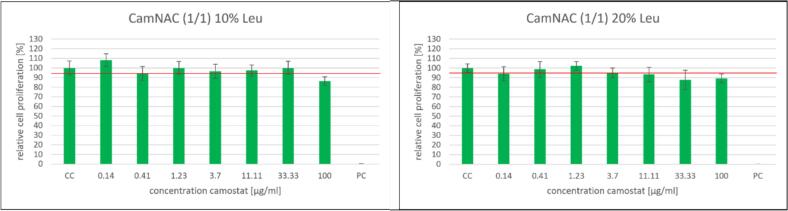
The graphs illustrate the results of a BrdU assay to determine cell proliferation of two dry powder formulations at different concentrations. The red line indicates 95% cell proliferation compared to the negative control. Left: CamNAC (1/1) 10% Leu composed of 63.85% camostat, 26.15% NAC and 10% L-leucine; right: CamNAC (1/1) 20% Leu composed of 56.75% camostat, 23.75% NAC and 20% L-leucine. (For interpretation of the references to colour in this figure legend, the reader is referred to the web version of this article.)

The 10% leucine formulation exhibits no cytotoxic effects up to a camostat concentration of 33.33 μg/mL. At the highest drug concentration of 100 μg/mL, cell proliferation drops to just under 90%. In contrast, the 20% L-leucine formulation shows a proliferation value below the 95% cell proliferation limit of the untreated cell control sample at a concentration of 3.7 μg/mL, albeit only narrowly at 94.94%. At a concentration of 11.11 μg/mL, the cell proliferation rate was 93.23%. However, as the concentration of camostat increased, the cell proliferation rate decreased to 89.20% at the highest concentration (Fig. 9).

As confirmed for cell viability, high concentrations also negatively affect cell proliferation. Even at lower concentrations, the 20% leucine sample inhibits cell growth and proliferation. This could be due to the difference in L-leucine concentration between the two formulations. Studies conducted *in vitro* have demonstrated that high concentrations of leucine can lead to cellular damage in toxic conditions, which may be worsened by DNA breaks (da Luz Dias et al., 2018). However, it should be noted that the IC_50_ values of the two formulations are at least 100 times lower than the concentration that negatively affects cell proliferation. To address this, options such as reducing the camostat or L-leucine content or decreasing the total dose of the dry powder formulation per inhalation could be considered.

### 3.9 Antiviral inhibition

**Fig. 10:**
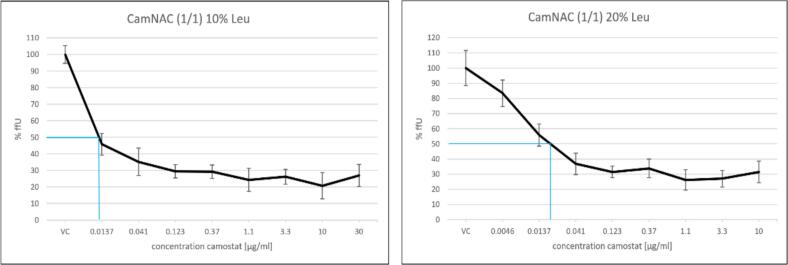
Graph of a virus inhibition assay (SARS-CoV-2 pseudoviruses, GFP reporter), virus inhibition was shown as a reduction of the relative focus forming unit with increasing camostat concentration. Left: CamNAC (1/1) 10% Leu composed of 63.85% camostat, 26.15% NAC and 10% L-leucine; right: CamNAC (1/1) 20% Leu composed of 56.75% camostat, 23.75% NAC and 20% L-leucine.

To evaluate the antiviral inhibitory effect of camostat in dry powder formulation, we used SARS-CoV-2 pseudoviruses that were equipped with a GFP reporter gene. HEK293T cells stable overexpress ACE2 and TMPRSS2 receptors (HEK293T/ACE2-TMPRSS2) were used. Infected cells were identified by GFP expression. The test result was expressed as a percentage of fluorescence forming units (ffU%), which represents the number of infected fluorescent cells in relation to the untreated virus control (VC) (Fig. 10).

Both graphs confirm that the serine protease inhibitor camostat has a strong effect on viral inhibition of SARS-CoV-2 pseudoviruses. The relative ffU%, which represents the proportion of infected cells expressing the fluorescent protein (GFP), decreases as the concentration of camostat increases. The formulation with a 10% leucine content has an IC_50_ value of <0.0137 μg/mL. In comparison, the formulation with a leucine content of 20% has an IC_50_ value of 0.019 μg/mL, which is higher than the formulation with a lower leucine content. The concentrations of the dry powder formulation used for the assay are adjusted to match the camostat concentration, and the NAC content is always equimolar to it. The two samples differ only in their L-leucine content. This assay suggests that a higher proportion of L-leucine has a negative effect on the antiviral efficacy of the formulation. It was not possible to establish a correlation as no literature on this effect was found. However, this might be due to the hydrophobicity of leucine interfering solubility but also the surface-active properties adsorbing to cellular surfaces. Overall, the results are in the range of the control experiment using camostat mesylate exhibiting IC_50_ values in the range from 0.24 to 0.27 μM (Fig. S1).

One inhalation dose corresponds to a capsule content of 20 mg of dry powder formulation. Depending on the formulation, the capsule contains either 12.77 or 11.35 mg of pure camostat. The human lung has a limited total volume of lung fluid, which is approximately 10–30 mL (Patton et al., 2010). Assuming that the formulation is evenly distributed, and the average lung fluid volume is 20 mL, the resulting concentrations would be 0.64 and 0.57 mg/mL, respectively. In terms of the respective IC_50_ values, this concentration is exceeded by a factor of approximately 47,000 and 25,000 at the stated dose. Considering the determined FPF values (ca. 55%), the lung concentration would drop to 0.35 or 0.31 mg/mL, which would still correspond to an exceedance of the IC_50_ by a factor of 14,000 to 26,000. This implies that also lower doses would be sufficient to achieve an antiviral effect. Neither the lung fluid nor the inhaled amount of active ingredient is evenly distributed in the lungs. It is also assumed that the entire formulation is applied in the lungs and dissolves in the entire lung fluid. However, despite these assumptions and simplifications, at these low IC_50_ values, it is clear that viral inhibition is very efficient by applying 20 mg of the dry powder formulations. Overall, the values of 0.02 μM and 0.048 μM for leucine 10% and 20% respectively, are below the values described by Hoffman et al. for Calu3 cells (IC_50_ ∼ 1 μM) (Hoffmann et al., 2020) which might be due to the cell line. We used HEK293 cells overexpressing ACE2 und TMPRSS2 for our experiments. In addition, Calu3 cells were found to be harder to infect than those cells explaining the lower IC_50_ values.

## 4 Conclusion

The objective of this study was to develop an inhaled dry powder formulation to potentially combat the respiratory SARS-CoV-2 virus. This approach can be most probably extended to other interesting pulmonary relevant viruses (i.e., SARS-CoV-1, MERS, NIPAH). For this, camostat mesylate, a known serine protease inhibitor, was converted into its base and then successfully spray-dried with NAC, providing mucolytic activity also known beneficial for the treatment of COVID-19 (Alam et al., 2023; Avdeev et al., 2022; Ibrahim et al., 2020; Shi and Puyo, 2020).

The aerodynamic properties and storage stability of the obtained microparticle dry powder were significantly improved by adding L-leucine as excipient. Increasing the L-leucine content resulted in a decrease in both MMAD and GSD. With 10% L-leucine the fine particle fraction of the formulation already reached a value above 50%. The reduction in geometric and aerodynamic diameter can be attributed to the formation of a hydrophobic leucine shell. This results in a decrease in interparticle agglomeration and a lower interaction with atmospheric ambient water, increasing storage stability. Powder X-ray diffraction experiments confirmed nano-crystallinity of the leucine shell and the amorphousness of the matrix composed of the two active ingredients, camostat and NAC. The rapid and complete dissolution of the microparticle formulation under lung-like conditions is due to the amorphous state of the active ingredients and the complex formation as described earlier (Günday Türeli et al., 2016; Lababidi et al., 2020; Lababidi et al., 2019). To evaluate the potential effect for application, the biological activity of the drug formulation, cell viability, cell proliferation, and antiviral efficacy were also tested using an *in vitro* pseudoviral set up. Antiviral activity was observed at very low concentrations. However, a negative effect on cell viability and cell proliferation was also observed at higher concentrations of camostat. Camostat concentrations of 100 μg/mL resulted in a significant decrease in cell viability and proliferation. This effect was significantly more pronounced for cell proliferation than for the metabolic activity of the cells. As those values are far above the activity threshold and the determined IC_50_ values, this should not lead to any limitation. In the case of cell proliferation, the effect was more pronounced for the formulation containing 20% L-leucine. The hydrophobicity of leucin might be the reason for this effect as speculated already in literature (Stella et al., 2024).

The developed inhaled dry powder formulation thus represents an innovative approach that allows local application of the drug at the point of entry of the virus. The powder matrix might allow for incorporation other compounds and active ingredients. Overall, this work not only offers a promising approach for local treatment, but also opens the door for further developments in the field of inhalable drugs to combat respiratory infections. Besides these very positive *in vitro* pre-evaluations the formulation would need to be tested for its application potential to underline the hypothesis of an improved therapeutical outcome using local drug application via inhalation.

## CRediT authorship contribution statement

**Justin Stella:** Writing – original draft, Visualization, Investigation. **Anja Germann:** Writing – review & editing, Methodology, Formal analysis. **Oliver Janka:** Writing – review & editing, Investigation, Formal analysis, Data curation. **Sylvia Wagner:** Writing – review & editing, Supervision, Resources, Conceptualization. **Marc Schneider:** Writing – review & editing, Supervision, Resources, Project administration, Conceptualization.

## Declaration of competing interest

The authors declare the following financial interests/personal relationships which may be considered as potential competing interests: Marc Schneider and Sylvia Wagner report financial support was provided by Saarland Ministry of Finance and Research. Oliver Janka reports financial support was provided by German Research Foundation. If there are other authors, they declare that they have no known competing financial interests or personal relationships that could have appeared to influence the work reported in this paper.

## Data Availability

Data will be made available on request.

## Appendix A Supplementary data

Supplementary material Image 1
