# Supplementary material for: Development of an inhalable dry powder formulation for inhibition of SARS-CoV-2
Source: Int J Pharm X. 2025 Jun 14;10:100346. doi: 10.1016/j.ijpx.2025.100346 (PMC12221515; doi:10.1016/j.ijpx.2025.100346)
Supplement: Supplementary file 1 — Supplementary material [file mmc1.pdf]

Supporting Information

Development of an inhalable dry powder formulation for inhibition of SARS-CoV-2

Justin Stella[a], Anja Germann[b], Oliver Janka[c], Sylvia Wagner[b], Marc Schneider[a,*]

[a] Department of Pharmacy, Biopharmaceutics and Pharmaceutical Technology, Saarland University, Campus C4 1, 66123 Saarbrücken, Germany

[b] Fraunhofer Institute for Biomedical Engineering IBMT, Department Bioprocessing & Bioanalytics, Joseph-von-Fraunhofer-Weg 1, 66280 Sulzbach, Germany

[c] Inorganic Solid-State Chemistry, Saarland University, Campus C4 1, 66123 Saarbrücken, Germany

Corresponding author *:

Marc Schneider, Biopharmaceutics and Pharmaceutical Technology, Saarland University, 66123 Saarbrücken, Germany

Email: Marc.Schneider@uni-saarland.de

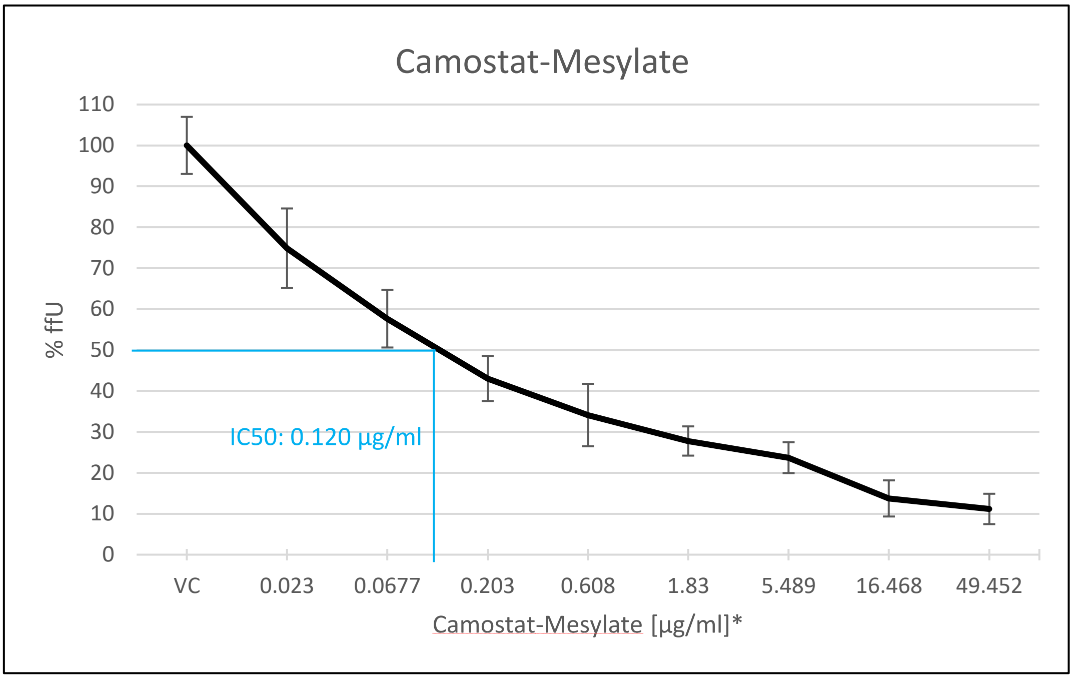

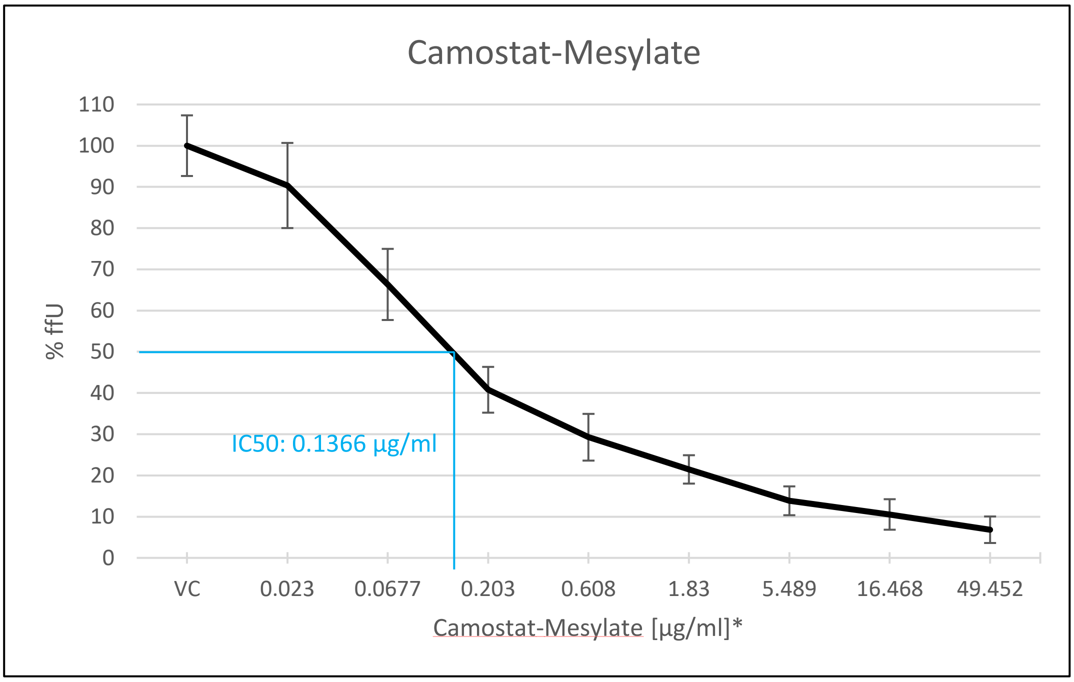

Figure S1 two examples of control experiments with camostat mesylate to evaluate the principal functionality of the used assay.

The assay was originally evaluated for its activity using camostat mesylate. The extracted IC_{50} values are in a range of 0.12 µg/mL to 0.1366 µg/mL corresponding to 0.24 µM and 0.27 µM respectively.
